# Laparoscopic Davydov’s Colpopoiesis for a Case of Mayer-Rokitansky-Kuster-Hauser (MRKH) Syndrome

**DOI:** 10.7759/cureus.13974

**Published:** 2021-03-18

**Authors:** Subha R Samantray, Ipsita Mohapatra, Nikku Harshini

**Affiliations:** 1 Obstetrics and Gynecology, All India Institute of Medical Sciences, Kalyani, Kalyani, IND; 2 Obstetrics and Gynaecology, Prathima Institute of Medical Sciences, Karimnagar, IND

**Keywords:** mayer-rokitansky-kuster-hauser (mrkh) syndrome, davydov’s colpopoiesis, vaginoplasty, laparoscopic davydov procedure

## Abstract

Congenital absence of vagina and uterus has been eponymously called Mayer-Rokitansky-Kuster-Hauser (MRKH) syndrome. The vagina may appear as a dimple with the presence of rudimentary uterine primordia and normal functioning ovaries. Its incidence is 1 in 4500 to 5000 female. Patients present with primary amenorrhea, normal external genitalia, and well-developed secondary sexual characteristics. Davydov’s colpopoiesis is one of the methods of vaginoplasty using the patient’s own peritoneum as a graft to line the neovagina. We present here a case of MRKH Syndrome where a laparoscopic Davydov procedure was chosen for vaginal reconstruction.

## Introduction

Congenital absence of vagina and uterus has been eponymously called Mayer-Rokitansky-Kuster-Hauser (MRKH) syndrome. The vagina may appear as a dimple with the presence of rudimentary uterine primordia and normal functioning ovaries. Its incidence is 1 in 4,500 to 5,000 female [1]. This rare anomaly of the female genital tract is caused by embryologic underdevelopment of the Mullerian duct with resultant vaginal and/or uterine agenesis [2]. Patients present with primary amenorrhea, normal external genitalia, and well-developed secondary sexual characteristics with normal female phenotype and karyotype of 46XX [3-5]. Investigations include 3D ultrasonography and a hormonal assay. About 6%-10% of patients present with cyclic abdominal pain associated with functional uterine rudiments, which can be assessed by an MRI [2]. Fifty-three percent of cases with MRKH syndrome have associated congenital anomalies, most of which are renal and musculoskeletal and can be best diagnosed by an MRI [6].

Davydov’s colpopoiesis is one of the methods of vaginoplasty using the patient’s own peritoneum as a graft to line the neovagina. We present here a case of MRKH Syndrome where a laparoscopic Davydov procedure was chosen for vaginal reconstruction.

## Case presentation

**Figure 1:**
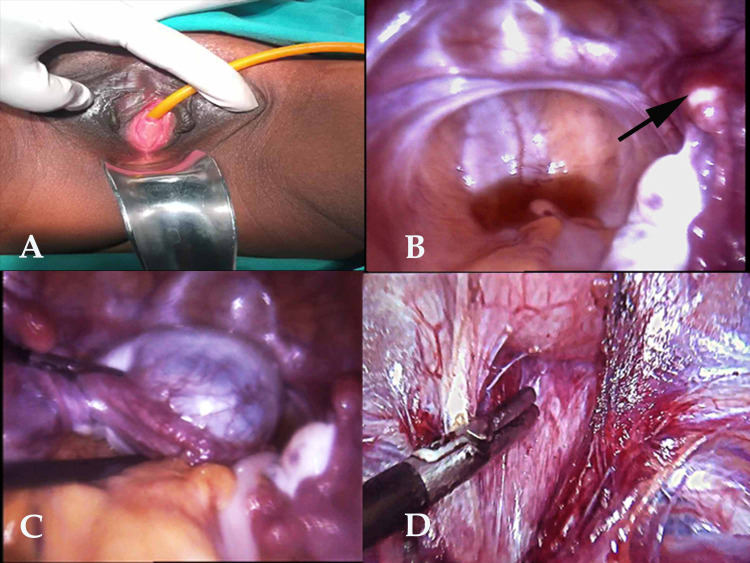
A: Blind ending vagina; B: internal view showing uterine anlagen (black arrow) with normal right ovary; C: left ovarian torsion; D: Rectovesicular space dissection.

A 23-year-old unmarried female presented with primary amenorrhea and intermittent left iliac fossa pain for three days. There was no history of cyclical abdominal pain, urinary symptoms, hoarseness of voice, or musculoskeletal complaints. There was no history of visual disturbance, secretion of milk from the breast, and no heat or cold intolerance. Her BMI was 17.94 kg/m^2^. The patient had no acne, hirsutism, or stria. Her spine and gait were normal and her vitals were stable. Secondary sexual characteristics were appropriate for age. There was no palpable mass per abdomen and no inguinal swellings. Gynecological examination revealed a grossly normal vulva and a blind-ending vaginal dimple 2 cm in length with patent external urethral meatus (Figure 1A). A good tone of anal sphincter was noted on a digital per rectal examination with no palpable uterus and cervix.

Laboratory evaluation revealed a normal hormonal assay and the karyotype was found to be 46XX. 2D Ultrasound revealed an absent uterus with a left ovarian simple cyst (6 x 5 cm) and a normal-sized right ovary. Bilateral kidneys were normal. No other concomitant defects were identified. MRI confirmed the diagnosis suggesting complete uterine agenesis with bilateral rudimentary uterine anlagen with no endometrial activity. The diagnosis of MRKH syndrome (Type-1) was made. 

The patient and her family were offered psychosocial counseling and all treatment options along with fertility issues were discussed. After informed written consent, Laparoscopic Davydov’s vaginoplasty was planned. She was placed in a lithotomy position under general anesthesia, a 12F Foleys catheter was placed in the bladder. The abdomen and vagina were approached simultaneously. Under the laparoscopic view, bilateral uterine anlagen with normal-sized right ovary, left ovarian simple cyst of 6 x 5 cm with torsion (ovarian vasculature not compromised) and bilateral normal fallopian tubes were noted (Figure 1). 

**Figure 2:**
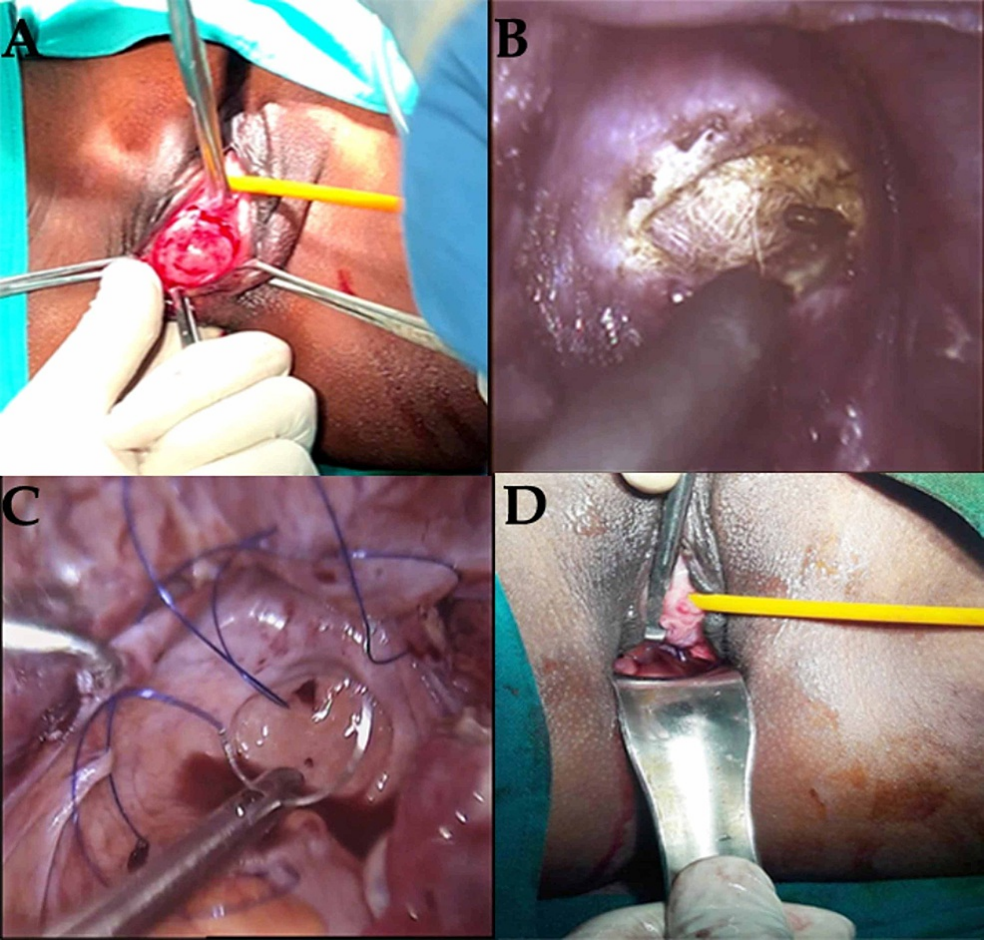
A: Vaginal dissection of rectovesicular space; B: incision of bridging tissue; C: formation of neovaginal apex; D: final postoperative view.

De-torsion followed by cystectomy and oophoropexy of left ovarian cyst was done. Laparoscopically transverse incision of 5cm was made in front of strands that connected the bilateral uterine anlagen. A free fold of peritoneum was created through gentle dissection. Under vision rectovesicular space was dissected. A transverse incision was made at the blind vaginal summit (Figure 2). Dissection of rectovesicular space was further done. Bridging tissue between the vagina and peritoneum was delineated by a sponge on swab and cut by monopolar electrosurgical hook. The mobilized peritoneum was pulled down and sutured to the edges of incised vaginal mucosa with the help of four Vicryl stitches. Apex of neovagina was made by closure of peritoneum with No 1-0 prolene, in a purse string manner, starting from peritoneum of bladder, left side round ligament, left side of utero-ovarian ligament , peritoneum between left ovary and rectum (with ureter under vision) and serosa of rectum and the same was repeated on other side.

A self-fabricated vaginal mold of size 10 × 3 cm using 10cc syringe, wrapped with strips of gauze, polyurethane condoms, and interceed was placed within the neovagina (secured with labial sutures) to maintain patency. On the seventh postoperative day, examination of neovagina and irrigation with normal saline was done under mild sedation. The mold was changed, which was removed subsequently after a week. A urinary catheter was kept in-situ for seven days. Self dilation, three times a day (20-30 mins) using lubricated Hegars dilators was advised. Post operative period was uneventful and the patient was discharged on 14th day with an achieved neovaginal length of 8 cm and 2 finger breadths. Clinical follow-up at 6months after surgery revealed patent neovagina of length 7.5 cm. The next follow-up was scheduled at 12 months following surgery. 

## Discussion

MRKH syndrome is a rare entity with absent middle and upper two-thirds of the vagina. Despite the absence of the uterus, fallopian tubes and ovaries are usually normal [7]. The majority of the patients with MRKH syndrome have complete aplasia of all Mullerian structures (Class U5/C4/V4 of ESHRE/ESGE Classification) and about 47%-84% have uterine remnants, either bi- or unilateral rudimentary horns with a cavity (Class U5a), or uterine remnants without cavity (Class U5b) [8,9]. Our patient belonged to ESHRE/ESGE class U5b/C4/V4. MRKH syndrome can be further classified as Type-I which includes isolated uterovaginal aplasia and that with accompanying defects constitute Type- II [10]. Concomitant defects include renal (20-50%), skeletal (50-70%) and rarely cardiac and digital abnormalities [2].

The exact etiology of this syndrome is uncertain. Some cases follow familial inheritance while a large number occur sporadically [11]. Patients typically present in adolescence with primary amenorrhea, well-developed secondary sexual characters, and an introital dimple. Cytogenetic analysis reveals 46XX karyotype. Differential diagnoses include imperforate hymen, androgen insensitivity syndrome, congenital adrenal hyperplasia, or Turner syndrome [1]. Our patient showed a normal endocrine balance. Noninvasive diagnostic aids include 2D/3D transabdominal ultrasonography and magnetic resonance imaging (MRI), out of which MRI can precisely delineate the anatomy and assess the presence of endometrium in the uterine remnants and concomitant defects [9].

The diagnosis of Mullerian anomalies has a profound psychological impact on fertility and sexuality. Psychosocial counseling and support are essential before the commencement of any intervention [1]. Several surgical and non-surgical methods of vaginal reconstruction have been outlined to retain sexuality and sexual function. Choice of procedure must be individualized which further depends upon individual need, surgeon's expertise, previous treatment attempts, and patient’s compliance [12]. Frank and Ingram dilation method remains the first line of intervention due to lower complication rates [2]. Other popular methods were Mc Indoe (split-thickness skin grafts) procedure, full-thickness skin grafts, and myocutaneous/fasciocutaneous flaps, William vulvovaginoplasty, conventional or laparoscopic Veicchetti traction procedure, Intestinal vaginoplasty, etc., moreover, each method has its own advantages and disadvantages.

The Davydov colpopoiesis is a method using the patient’s own peritoneum to epithelialize the neovagina [13]. It is a three-stage procedure that requires dissection of space between the urethra, bladder, and rectum, followed by peritoneal mobilization which is subsequently sutured to the edges of the vaginal vestibulum. It also requires postoperative dilation and is suitable for women who underwent extensive pelvic surgeries. It is one of the simplest, safest, and quickest methods with many advantages including good lubrication with resultant satisfactory intercourse, lack of granulation tissue, and absent scar formation [14]. Patients comparatively have less bleeding, less postoperative pain, shorter stay, quick recovery, and satisfactory cosmetic outcome although ascending infections are reported in few cases [9, 14].

The outcomes are assessed in the form of anatomic and functional success. Anatomic success is defined in terms of achieved neovaginal length of at least 6cm, whereas functional success is the ability to have a complete satisfactory sexual intercourse which can be assessed by the Female Sexual Function Index [FSFI] questionnaire which takes into account parameters like desire, arousal, lubrication, orgasm, satisfaction and pain during intercourse [9]. Although Frank and Ingram's vaginal dilatation is considered a non-invasive and first-line intervention for the creation of a neovagina, the anatomical and functional success rate for surgical vaginoplasty was observed to be more than Frank^'^s dilatation (90%-96% vs. 70%-78%) [9].

A surgical procedure on our patient was planned as she expressed non-compliance with Frank dilation therapy. Davydov procedure was chosen for our patient because of its simplicity and safety as well as it seems to be an effective method of creation of a neovagina in patients with MRKH syndrome [14]. The procedure was performed with no intraoperative injury, minimal blood loss. An anatomical length of 9cm was observed at end of the procedure. The postoperative period remained uneventful. She was instructed for self vaginal dilation daily with the use vaginal dilator (10 × 3cm) and followed up for 6months after surgery. A good anatomical length of 7.5 cm was achieved. Further, follow-up was scheduled at 12 and 24 months to know the long-term outcome.

MRKH syndrome is a major cause of absolute uterine factor infertility. Possible fertility options include adoption and gestational surrogacy and live births following uterine transplantation have also been reported [1,14,15]. Newer emerging Organ Engineering Technology constitutes the culture of vaginal epithelial and smooth muscle cells and their incorporation on-to biodegradable scaffold to manufacture vaginal tissue in-vitro. It is considered a breakthrough in the field of regenerative medicine and can be used for surgical reconstruction of the vagina in patients with MRKH syndrome [16].

## Conclusions

Mullerian agenesis is a rare congenital anomaly that requires a multidisciplinary approach with mighty psychological assistance. Good communication with the patient and emotional resilience is a pre-condition for intervention. Non-surgical vaginal dilation remains the first-line therapy. Laparoscopic neovaginal reconstruction techniques are very alluring of which Laparoscopic Davydov procedure remains unscathed and effective. The potency of initial surgery and adequate postoperative management along with patient compliance influence the success rates. Adoption and surrogacy can be offered as fertility options. Current progress and future prospects of uterine transplantation are being contemplated with questionable feasibility and viability. Tissue-engineered autologous vaginal reconstruction, may, in the future, be an alternative.
